# Bidirectional Brain‐gut‐microbiota Axis in increased intestinal permeability induced by central nervous system injury

**DOI:** 10.1111/cns.13401

**Published:** 2020-05-29

**Authors:** Xiao‐jin Li, Xin‐yu You, Cong‐ying Wang, Xue‐li Li, Yuan‐yuan Sheng, Peng‐wei Zhuang, Yan‐jun Zhang

**Affiliations:** ^1^ Tianjin University of Traditional Chinese Medicine Tianjin China; ^2^ Tianjin State Key Laboratory of Modern Chinese Medicine Tianjin Key Laboratory of Chinese Medicine Pharmacology Tianjin China

**Keywords:** autonomic nervous system, central nervous system injury, enteric nervous system, hypothalamic‐pituitary‐adrenal axis, increased intestinal permeability

## Abstract

Central nervous system injuries may lead to the disorders of the hypothalamic‐pituitary‐adrenal axis, autonomic nervous system, and enteric nervous system. These effects then cause the changes in the intestinal microenvironment, such as a disordered intestinal immune system as well as alterations of intestinal bacteria. Ultimately, this leads to an increase in intestinal permeability. Inflammatory factors produced by the interactions between intestinal neurons and immune cells as well as the secretions and metabolites of intestinal flora can then migrate through the intestinal barrier, which will aggravate any peripheral inflammation and the central nervous system injury. The brain‐gut‐microbiota axis is a complex system that plays a crucial role in the occurrence and development of central nervous system diseases. It may also increase the consequences of preventative treatment. In this context, here we have summarized the factors that can lead to the increased intestinal permeability and some of the possible outcomes.

## 1 INTRODUCTION

**FIGURE 1:**
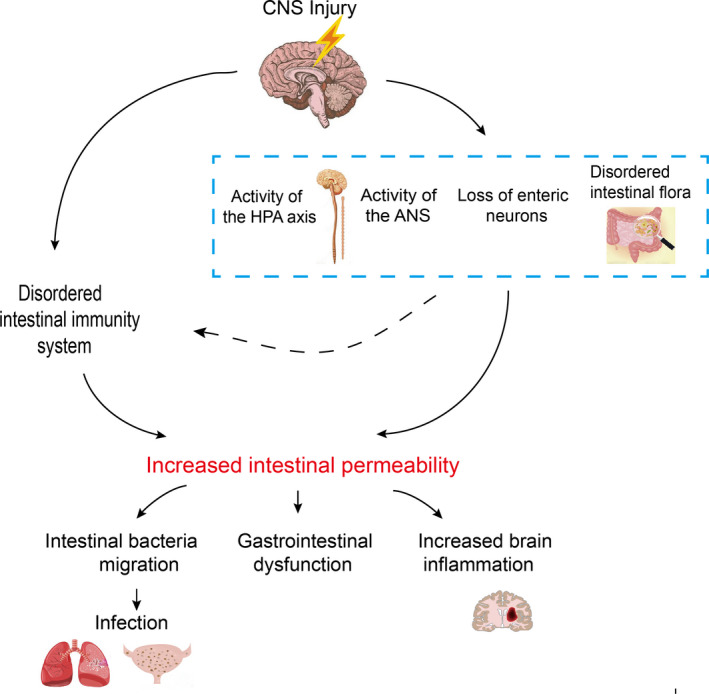
Excessive activation of the hypothalamus‐pituitary‐adrenal axis and the autonomic nervous system, as well as partial loss of intestinal neurons and imbalance of the intestinal flora after CNS injury, changes the internal environment, which can lead to increased intestinal permeability furtherly. These changes can lead to poor outcomes such as intestinal bacteria translocation, gastrointestinal dysfunction, and aggravation of brain injury. Following this, secondary infections, especially pulmonary and urinary tract infections, can occur. Nervous dysfunction and intestinal flora imbalances also result in intestinal immune imbalances, increasing intestinal permeability and worsening the patient's outcome

The hypothalamus‐pituitary‐adrenal (HPA) axis and the autonomic nerves are activated under the stimulus of inflammation induced by a central injury. The brain‐gut‐microbiota axis is the channel for information exchange between the brain and the intestine. The central sympathetic and parasympathetic nerve fibers transmit information to the intestine,^1^ and the intestine in turn affects the brain activity through the vagus nerve and the intestinal immune system.^2^ Activation of the vagus nerve and an increase in sympathetic nerve activity can lead to inhibition of immune cell function and eventually lead to intestinal inflammation.^3^ Additionally, the intestinal microorganism‐host interactions may play a crucial role in regulating intestinal and brain activity.^4^ Corticotropin‐releasing factor (CRF) released by the HPA axis can also influence the intestinal function. It can also directly act to increase intestinal permeability. We believe that an increase in intestinal permeability is a key factor involved in the cross‐talk between intestinal and brain function. After a central nervous system (CNS) injury, not only will gastrointestinal dysfunction quickly appear, but over time, the CNS injury will also become aggravated and the patient's prognosis will worsen (Figure 1).

## 2 INCREASED PERMEABILITY OF THE INTESTINAL MUCOSAL BARRIER AFTER CNS INJURY

After CNS injury, the permeability of the intestinal mucosal barrier is increased. The destruction of the integrity of the intestinal wall and intestinal inflammation are the key factors involved in this effect. In the intestinal tract, the conduction of biochemical signals and digestive function depend on the regulation of the intestinal mucosal barrier and the high integrity of the tight junctions.^5^ In rodent models of CNS injury, several kinds of changes that impair the integrity of the intestinal mucosa have been observed,^6^, ^7^ such as damage to intestinal mucosal epithelial cells, apoptosis of intestinal epithelial cells, intestinal tissue villi loss, inflammatory cell infiltration, and changes in intestinal goblet cells.^8^, ^9^, ^10^ In addition, a lack of tight junction proteins is a critical reason for the increased intestinal mucosal permeability. Decreased expression of the tight junction proteins ZO‐1, occludin, claudin‐1, and claudin‐5 in the intestine accelerates the increase in intestinal of mucosal permeability after CNS injury.^8^, ^11^, ^12^ Apoptosis of intestinal epithelial cells and the loss of tight junctions between adjacent intestinal cells in a traumatic brain injury (TBI) rat model were observed under electron microscopy.^13^ Mucin secreted by goblet cells has a protective effect on the intestinal mucosa, so damage to the intestinal mucosa is associated with a decrease in the number or function of goblet cells. Intestinal inflammation that alters intestinal contraction function significantly affects the permeability of the intestinal mucosal barrier.^14^, ^15^ Enhancement of NF‐KB activity and the production of the proinflammatory cytokines IL‐1α, IL‐1β, and IL‐17 in the intestine can lead to the delayed contractile activity of the ileum.^16^ Increased expression of TNF‐α in the intestinal tract after TBI has been demonstrated.^14^ The structure of the tight junction proteins in intestinal epithelial cells depends on the production and regulation of TNF‐α. Overexpression of TNF‐α promotes increased permeability of the intestinal mucosal barrier.^11^

## 3 FACTORS INDUCING INCREASE OF INTESTINAL MUCOSAL PERMEABILITY AFTER CNS INJURY

### 3.1 Enhanced activity of the HPA axis induced by CNS injury

Enhanced activity of the HPA axis after CNS injury releases many hormones, which can influence the intestinal mucosal permeability. Vascular ischemia and hypoxia in the injured tissue within hours of CNS injury will lead to massive apoptosis and necrosis, which will induce innate and adaptive immune responses. In addition, necrotic cells release danger‐associated molecular patterns,^17^, ^18^ which activate pattern recognition receptors. Inflammation will develop, followed by the release of proinflammatory cytokines by nearby microglia and macrophages.^17^, ^19^ The inflammatory factors will then activate the neural circuit and trigger the activity of the HPA axis.^20^ The hypothalamus releases hormones that will act on the pituitary gland to induce the secretion of adrenocorticotropic hormone, which then promotes the production and release of glucocorticoids through the zona fasciculata of adrenal cells.^21^, ^22^, ^23^ The increased level of adrenocorticotropic hormone leads to damage to the intestinal mucosal.^24^ Activation of cerebral corticotropin‐releasing factor (CRF)‐R2 subtype inhibits gastric motility, while CRF‐R1 stimulates neuromotility of the colon and induces visceral hypersensitivity. Intestinal CRF‐R1 activates colonic myenteric neurons and induces the secretion of serotonin by mucosal cells. Mucus and prostaglandin E2 induce the degranulation of mast cells to enhance mucosal permeability and propel motor function.^25^ A recent study suggested that in the mice with acute spinal cord injury (SCI), the release of serum norepinephrine was inhibited, which occurred concomitantly with cortisol level increased.^21^ The authors of another study also reported that the levels of cortisone, corticosterone, epinephrine, and corticotropin‐releasing hormone were increased in the plasma of mice after cerebral ischemia.^26^ In addition, studies of clinical cases showed that the increased release of cortisol in humans under stress results in an increase in permeability of the small intestine. After administration of exogenous corticotropin‐releasing hormone, both the release of cortisol and the intestinal permeability are increased, along with activation of mast cells.^27^ Experiments have shown that exogenous CRF enhances the intestinal permeability and affected the function of the gastrointestinal tract.^28^ Under stress, the increase in cortisol and the activation of mucosal mast cells cause peripheral CRF receptors to participate in the changes in intestinal function.^28^ After CNS injury, glucocorticoids are released, which increases the intestinal epithelial permeability by disrupting the function of the intestinal epithelial mucosa.^29^, ^30^ Likewise, the number of mast cells in the gut will decrease because of the increased levels of glucocorticoids.^31^ Mast cells are essential for producing host antimicrobial agents and are involved in intestinal mucosal regulation, epithelial secretion, and the contractions of intestinal smooth muscles. Therefore, an increased glucocorticoid level may be one of the factors involved in gastrointestinal dysfunction after CNS injury.

### 3.2 Disordered autonomic nerves system induced by CNS injury

The sympathetic and parasympathetic nerves are activated immediately after CNS injury, releasing catecholamines and acetylcholine into the peripheral circulation.^26^, ^32^ Some neurotransmitters, such as catecholamines and acetylcholine, indirectly regulate the intestinal mucosal permeability. Modulated by the CNS, the autonomic nervous system communicates with the enteric nervous system (ENS), which regulates host physiological homeostasis. The regulation of gastrointestinal blood flow through sympathetic vasoconstriction is neuro‐dependent and relies on α‐adrenergic receptor antagonists to reduce vasoconstriction.^33^ Excessive norepinephrine in the blood has a negative effect on gastrointestinal vasoconstriction, and an increase in some endogenous vascular substances also leads to a decrease in intestinal blood flow, which will cause gastrointestinal mucosal damage.^34^, ^35^ It has been reported that cutting the sympathetic nerves in cerebral ischemia rats can increase the occurrence of stress ulcers.^24^ Sympathetic nerve branches also regulate the number, degranulation, and activity of mast cells, and can lead to an imbalance in histamine release from tryptase and induce stress‐related muscle dysfunction.^36^

The brain and gastrointestinal tract also communicate through the vagus nerve. The vagus nerve fibers can innervate the myenteric plexus in the colon and ileum. After the central nervous system injury, the vagus nerve becomes activated and enhances the secretion of gastric acids through the discharge of acetylcholine from its distal end. Moreover, the vagus nerve impulses can directly stimulate gastric parietal cells to secrete acid and indirectly stimulate gastric antral G cells to release gastrin. The increase in H+ ions can promote excessive histamine secretion and increase gastrointestinal permeability.

### 3.3 Loss of enteric neurons induced by CNS injury

The sympathetic and parasympathetic nerves that enter the inner wall of the intestinal tract form synapses with the enteric nervous, transmitting information from the CNS. The enteric nervous system is composed of the gastrointestinal submucosal nerve plexus, intermuscular nerve plexus, and interconnecting fibers. It mainly regulates the peristalsis of the gastrointestinal tract, changes in blood flow, and the secretion of water and electrolytes.

Loss of enteric neurons after a central nervous injury is another factor that can increase intestinal mucosal permeability. It has been revealed in the literature that patients with spina bifida and spinal cord injuries may show signs of the loss of colonic neurons loss, decreased nerve fiber density, and severe loss of interstitial cells of Cajal (ICC) around the myenteric plexus.^37^ It was also reported that in mice with permanent middle cerebral artery embolization (pMCAO) both intestinal submucosal neurons and intermuscular neurons were lost. The loss of neurons was especially severe in the ileum, where the maximum loss was observed at 3 days after stroke, and the number of intestinal neurons was decreased in the colon as well.^38^, ^39^ Quantitative analysis of subsets of specific submucosal neurons in the ileum of stroke mice demonstrated that the submucosal cholinergic ChAT+ cells were lost.^40^ Changes in the number and structure of intestinal neurons affect intestinal function.^40^ The intermuscular nerve plexus in the enteric nervous system plays a key role in regulating the intestinal motility, and the submucosal plexus regulates mucosal secretions and blood flow.^2^ Activation of α2‐adrenergic receptors in the myenteric plexus inhibits gastrointestinal motility, whereas activation of α2‐adrenergic receptors on submucosal neurons reduces mucosal electrolyte secretion.^33^ Loss of the intestinal myenteric plexus weakens the peristaltic intestinal function and causes symptoms such as constipation.^37^

### 3.4 Imbalanced intestinal flora induced by CNS injury

Increased intestinal permeability is related to changes in the intestinal flora composition and its metabolism.^41^, ^42^ Intestinal barrier dysfunction and intestinal movement disorders in poststroke mice are related to decreased diversity of intestinal microbial species and excessive bacterial growth.^43^ The changes of firmicutes, bacteroides, and actinomycetes in the intestinal tract of cerebral ischemia mice model are significant.^43^ Changes in the variety and magnitude of intestinal microorganisms aggravate inflammation in the intestinal tract, and vice versa.

Gut microbiotas interact with neurons to influence intestinal permeability. Enhanced autonomic nerve activity will increase norepinephrine content and induce the transformation of intestinal flora.^10^, ^44^, ^45^ It has been shown that the microbiota can affect the activity of the enteric nervous system by producing molecules that act as local neurotransmitters. Reduced intestinal transport that is controlled by parasympathetic activity is associated with overgrowth of the small intestine bacteria and increased bacterial translocation.^46^ Additionally, microbiota‐host interactions in the gut lead to the release of microbial byproducts (cytokines, chemokines, neurotransmitter) that can infiltrate the blood lymphatic systems and the brain.^47^ Some bacteria can release endotoxins or short‐chain fatty acids, acetylcholine and other substances to stimulate intestinal reactions.^47^, ^48^, ^49^ Harmful bacteria in the intestine can bind to the surface molecules of intestinal epithelial cells to disrupt the expression of tight junction proteins, leading to intestinal mucosal barrier damage. Probiotics can improve the inflammation in the intestinal tract and reduce intestinal permeability, resulting in the protection of the intestinal mucosa.^50^

### 3.5 Disordered intestinal immunity system

After CNS injury, ileal mucosal injury can activate intestinal immunity. Impaired immune cell function contributes to the release of proinflammatory factors and aggravates the intestinal damage. The intestinal immune system is composed of Peyer's patches, individual immune cells within the intestinal epithelium and the lamina propria. The system is controlled by sympathetic nerves, and it forms an immune barrier between the intestinal microflora and the systemic circulation.^51^

Immune dysfunction of the intestinal tract is characterized by changes in the quantity and activity of intestinal immune cells and the abnormal secretion of inflammatory factors by immune cells. However, there are different conclusions about the changes in the quantity of immune cells in intestinal‐associated lymphoid tissues after CNS injury. Liu Yanning and other researchers found that T lymphocytes, especially in CD4+ and CD8+ T cells, in Peyer's plaques increased significantly at 12 and 24 hours after cerebral ischemia. However there were no remarkable differences found for B lymphocytes and intraepithelial lymphocytes.^6^ In contrast, studies by Dragana Stanley have shown a notable increase of B cells in the mesentery 24 hours after cerebral ischemia, particularly of IgA + B cells.^40^ Another study showed that 24 hours after cerebral ischemia, both the B cells and the T cells in Peyer's patches were decreased,^52^ whereas there was no difference in the natural killer cells, macrophages, or lymphocytes subsets in the intestinal epithelium and lamina propria.^53^ In addition, compared with the sham‐operated group, the proinflammatory factors IL‐17 and IFN‐γ were increased 4‐fold in the Peyer's patches of cerebral ischemic mice.^43^

There are many factors that contribute to the intestinal immune system disorders that can increase intestinal mucosal permeability. In the following sections, we will discuss some of the more important factors to consider.

#### 3.5.1 Autonomic nerve‐induced intestinal immune disorder

Dysfunction of autonomic nerves induces a disordered intestinal immune system. The neurotransmitters released by the autonomic nerve affect the activity of intestinal macrophages, and the release of acetylcholine from the vagus nerve indirectly inhibits the activity of macrophages.^54^ Bone morphogenetic protein 2 secreted by muscle macrophages regulates the activity of intestinal neurons and affects smooth muscle contractility under steady‐state conditions. After TBI, the expression of glial fibrillary acidic protein, a marker of glial activity in the intestine, is increased. Furthermore, glial fibrillary acidic protein is expressed after stimulation of the vagus nerve, indicating that stimulation of the vagus nerve can enhance the activity of glial cells.^7^ Glial GFAP and Sox10 in the colon are notably increased at 28 days after TBI injury.^55^ Intestinal glial cells play an important role in intestinal integrity and are activated in response to intestinal inflammation. Activated glial cells can produce abundant proinflammatory factors and aggravate the injury to the intestinal mucosa.^7^

#### 3.5.2 Intestinal immune disorder induced by the loss of enteric nerves

After CNS injury, in addition to the release of abnormal neurotransmitters by the intestinal sympathetic and parasympathetic nerves, there is also a loss of neurons in the intestinal tract that may affect the activity and homeostasis of macrophages. As described above, studies have found that the loss of intestinal subcholine cholinergic neurons results in a decrease in the number of cholinergic ChAT+ cells in the submucosal layer, which can stimulate the inflammatory immune response in the gut.^40^ The loss of VIP and nNOS neurons that control intestinal blood flow and electrolyte secretion may lead to defective cell function.^38^, ^56^

#### 3.5.3 Increased expression of intestinal TREM1 induces intestinal immune disorder

An emerging line of evidence has shown that the expression of triggering receptors in myeloid cells 1 (TREM1) in the Mo/MΦ subset in the propria of the small intestine increases at 4.5 hours after middle cerebral artery occlusion (MCAO). Activation of TREM1 further exacerbates the sympathetic nerve‐dependent intestinal permeability and induces the migration of LPS and bacteria into the surrounding tissues. There are a large number of macrophages in the intestinal lamina propria, which can maintain a complete intestinal epithelial barrier and play a role in antiinflammatory homeostasis. TREM1, which is expressed on myeloid lineage cells, is an amplifier of proinflammatory innate immune responses. This study also demonstrated that compared with sham mice, FITC‐dextran serum levels were increased in wild‐type mice who were administered scrambled peptide after MCAO, but the FITC‐dextran levels were markedly reduced in Trem1−/− mice. Morphometric examination of the lamina propria of the jejunum and ileum revealed decreases in the width of the muscularis layer and crypt height in Trem1+/+ mice. Additionally, expression of the epithelial cell adhesion molecule (EpCAM) was reduced in wild‐type mice, but not in Trem1−/− mice. These results suggest that activated TREM1 further increases intestinal permeability and bacterial translocation after MCAO.^57^

#### 3.5.4 Intestinal immune disorder induced by imbalanced intestinal flora

Intestinal flora imbalances also alter the immune homeostasis of the small intestine. The interface between the intestinal lumen and mucosal is where the host interacts with intestinal bacteria. Molecular exchanges through the epithelium and the mucus layer promote communication between the gut and the immune system.^47^ The gut microbiota influences the populations and function of subsets of immune cells. Intestinal microbiota can activate Toll‐like receptors on intestinal immune cells and promote the release of the inflammatory cytokines IL‐1β, IL‐6, IL‐8, and TNF‐α.^58^ One study showed that bacterial disorders lead to an upregulation of regulatory T cells and a downregulation of IL‐17–positive γdT cells by altering dendritic cell activity.^59^ The number of mucosal CD11b monocytes was increased in mice vaccinated with feces of focal MCAO.^43^ The state of the gut microbiota affects the polarization of naive T cells in the gut, and the antiinflammatory microbial group induces polarization of naive T cells in the lamina propria toward antiinflammatory Tregs.^60^

### 3.6 Abnormal secretion of brain‐gut peptide after CNS injury

Both autonomic and enteric nerves secrete cholinergic neurotransmitters, adrenergic neurotransmitters, and neuropeptides such as motilin, vasoactive intestinal peptide, and substance P into the blood to regulate gastrointestinal activity.^61^ After CNS injury, the abnormal secretion of brain‐gut peptides can damage the gastrointestinal mucosal layer.

The vagus nerve controls peripheral ghrelin secretion. Acetylcholine and norepinephrine can increase ghrelin secretion.^62^ After CNS injury, the increased secretion of acetylcholine and norepinephrine leads to an increase of blood ghrelin, which is one of the factors causing gastrointestinal mucosal damage.^12^ Vasoactive intestinal peptide (VIP) is widely distributed in the central, peripheral nervous system and surrounding tissues. The number of VIP‐immunoreactive submucosal neurons in the ileum is increased in the pMCAO model.^38^ Elevated levels of vasoactive intestinal peptides lead to excessive gastric acid secretion. Somatostatin has a strong inhibitory effect on gastric acid secretion. Decreased expression of somatostatin in the gastric antrum mucosa after spinal cord injury is also an important cause of damage to the gastrointestinal mucosa.^63^

## 4 OUTCOMES OF INCREASED INTESTINAL MUCOSAL PERMEABILITY AFTER CNS INJURY

### 4.1 Bacterial translocation

Cases of central nervous injuries are complicated by infectious diseases such as pneumonia and sepsis, which are consequences of bacterial migration. The intestinal single cell layer prevents bacteria from coming into contact with the visceral tissues, which is achieved by secretion of the mucus layer by epithelial goblet cells.^47^ Therefore, intestinal epithelial cell apoptosis and a decrease of goblet cells after central nervous injury may lead to increased mucosal permeability. Some harmful bacterium and their metabolites can migrate through the intestinal mucosal barrier to the internal organs, which conversely aggravates the central nervous system injury.^60^ For example, the number of microorganisms in the ileum and colon decrease after stroke while the number of corresponding microorganisms increases in the lungs.^40^ Great changes to the microbes occur in the gastrointestinal mucosa after 24 hours after stroke onset. There is an increased abundance of *Akkermansia muciniphila* and an excessive increase of Clostridium bolteae and Clostridium indolis, which are associated with gastrointestinal dysfunction, whereas overall the number of bacteria is reduced in the stroke mouse model gut.^64^ When the feces of poststroke mice were transplanted into the gut of SPF‐grade mice, some bacteria were found to increase in the blood, lung, liver, and mesentery of the latter.^40^

### 4.2 Dysfunction of the gastrointestinal gut

There has been an increase in the incidence of CNS injury diseases such as stroke, TBI, and SCI in recent years, and these patients often have a poor prognosis.^32^, ^65^ Numerous data from the clinic have shown that patients with CNS injuries are often accompanied by severe gastrointestinal dysfunction, which mainly manifests as inhibition of gastrointestinal motility and gastrointestinal mucosal damage.^35^, ^66^

Bidirectional neuroimmune circuits connect intestinal immune and neuronal cells and regulate fundamental aspects of enteric physiology. Mast cells can interact with nearby neurons. The histamine and tryptase produced by mast cells can activate submucosal neurons and peptidergic neurons to secrete VIP and SP, which can for regulate mast cells.^67^ After CNS injury, the immune system in the intestine is disordered and the mast cells release histamine, which affects the function of the gastrointestinal tract.

Reduced parasympathetic nerve activity following acute CNS injury is associated with overgrowth of bacteria in the small intestine and increased bacterial translocation. Substances released by bacteria and metabolites, enteroendocrine factors, and media released by the gastrointestinal immune system can all affect gastrointestinal movement.^42^, ^49^

Bacterial endotoxins such as lipopolysaccharide may affect bowel movements. For example, gastric emptying can be delayed by it affecting the enteric nervous system and related transmitters (such as nitric oxide).^48^ Increased Clostridium bolteae and Clostridium indolis are associated with gastrointestinal dysfunction.^61^

### 4.3 Aggravation of central nervous system damage

Numerous experiments have shown that increased intestinal permeability promotes intestinal flora translocation, and this aggravates cerebral infarctions by stimulating the innate and adaptive immunity after CNS injury.^68^ Transplanting feces of mice affected by stroke into sterile mice can increase the cerebral infarct size and aggravate functional defects in the sterile mice.^43^ Additionally, the mRNA levels of IFN‐(γ) and IL‐17 were increased significantly in the brain of sterile mice that had received cecal contents from MCAO mice.^60^ The number of microglia/macrophages increased in the ischemic hemisphere of mice colonized by bacteria, and the morphology of the microglia in the contralateral hemisphere was also affected. There was upregulation of the expression of the proinflammatory cytokines IL‐1 and TNF‐α associated with microglia activation.^69^ Moreover, there were excessive numbers of IL‐17 + γδT cells in the intestinal lamina propria and epithelial cells, which can aggravate ischemic brain injury by secreting IL‐17, an inducer of chemokines, and generating catalytic signals from peripheral myeloid cells. After MCAO, disturbance of the intestinal flora results in a decrease in IL17 + γδT in the intestine. However, L17 + γδT will accumulate in the meninges. IL‐17 + γδT cells are associated with the infiltration of neutrophils and monocytes into the brain parenchyma and they can aggravate brain damage.^59^ In the spleen of bacterial colonized mice, not only T cells and B cells, but also CD4+, FOXP3+, and Th17 in the intestinal immune region of Peyer's patches and specific T‐helper cell subtypes including IL‐10 and IL‐17 in the ischemic brain are increased.^69^ Interestingly, increased permeability of the intestinal mucosal barrier can promote the migration of TREM1. The increase in TREM1 PET signals may reflect the induction of TREM1 and the response to pathogen‐associated molecular model molecules (PAMP) migrating across the intestinal barrier after MCAO. The expression of Mo/MΦ subsets in the spleen and blood as well as intestinal TREM1 is increased. Both TREM1 and neutrophils infiltrate into the brain and increase the area of the cerebral infarction.^57^ TREM1 inhibits functions necessary for brain recovery, including antioxidant glutathione metabolism, antiinflammatory TREM2 signaling, and lysosomal degradation.

## 5 CONCLUSION

Many studies have demonstrated that dysfunction of the CNS seriously affects gastrointestinal tract function. In this work, we first explored the key elements that lead to impairment of the intestinal permeability induced by CNS injury based on the brain‐gut‐microbiota axis. We focused on the effects of increased intestinal permeability and analyzed the outcomes. We believe that the neurotransmitters and hormones released by the disordered autonomic nerves and the HPA axis after CNS injury are the primary factors leading to increased intestinal permeability. However, the neuro‐humoral‐immunity functions are complementary and interact, and this regulates intestinal biological signals and intestinal function. Intestinal inflammation caused by the intestinal nerves, disordered intestinal flora, and an imbalanced intestinal immune system are a factor directly leading to increased intestinal permeability. The destruction of the intestinal mucosal barrier and the increases in inflammatory factors are inseparable. The brain‐gut‐microbial axis is a complex system that has become a research hotspot in recent years.^47^ The increased intestinal permeability promotes the migration of intestinal flora and lipopolysaccharides, causing local organ infection and aggravating the CNS injury.^5^, ^57^, ^60^ There is no available therapy that can reduce the intestinal permeability. The administration of acid‐suppressing agents to patients after cerebral hemorrhage accompanied by gastric bleeding will increase the bleeding rate,^7^ and therefore, antacids are not suitable for the treatment of gastrointestinal diseases after CNS injury. Antiplatelet drugs are used to prevent gastric bleeding in patients with cerebral ischemia.^6^ Exogenous gastrointestinal hormones such as calcitonin gene‐related peptide (CGRP) and ghrelin have protective effects on gastrointestinal dysfunction after brain injury.^24^, ^65^ Current researches suggest that probiotics can reduce both intestinal inflammation and intestinal permeability, and thus provide simultaneous intestinal mucosa protection and cerebral protection.^16^, ^50^ One report showed that gut‐innervating TRPV1 + nociceptor sensory neurons in the dorsal root ganglion and vagal ganglia release CGRP to regulate the levels of microfold cells and segmented filamentous bacteria in Peyer's patches to resist the invasion of Salmonella enterica serovar Typhimurium into the internal organs. The loss of TRPV1 + DRG receptor neurons will change the type and quantity of intestinal flora; however, this does not directly effect on gut‐specific immune cells.^70^ Cross‐talk of nociceptor neurons with epithelial cells and intestinal microorganisms can be used to regulate nociceptor neurons with epithelial cells and intestinal microorganisms can be used to regulate the intestinal barrier defense. We speculate that the loss of TRPV1 + DRG receptor neurons is related to the destruction of the intestinal mucosal barrier and increased intestinal barrier permeability. Therefore, it is necessary to investigate whether gut‐innervating TRPV1 + nociceptor sensory neurons in the dorsal root and vagal ganglia are involved in intestinal flora disorder and intestinal barrier function damage caused by CNS injury. This is expected to be one of the targets for the treatment of gut dysfunction.

In addition, the results of numerous studies have suggested that the primary task of treating infection after central injury is to restrain the migration of intestinal bacteria into the body. Therefore, protecting the intestinal barrier is a priority after a CNS injury.

## CONFLICT OF INTEREST

The authors declare no conflict of interest.
